# Prognostic significance of concentric left ventricular hypertrophy at peritoneal dialysis initiation

**DOI:** 10.1186/s12882-021-02321-1

**Published:** 2021-04-16

**Authors:** Misato Tomura, Yoshifumi Hamasaki, Yohei Komaru, Yoshihisa Miyamoto, Ryo Matsuura, Akihiko Matsumoto, Kent Doi, Haruki Kume, Masaomi Nangaku

**Affiliations:** 1 Hemodialysis and Apheresis, The University of Tokyo Hospital, 7-3-1 Hongo, Bunkyo, Tokyo, Japan; 2 Urology, Yaizu City Hospital, Shizuoka, Japan; 3 Acute Medicine, The University of Tokyo Hospital, Tokyo, Japan; 4 Urology, The University of Tokyo Hospital, Tokyo, Japan; 5 Nephrology and Endocrinology, The University of Tokyo Hospital, Tokyo, Japan

**Keywords:** Concentric left ventricular hypertrophy, Peritoneal dialysis, Life prognosis, Cardiovascular disease, Serum albumin

## Abstract

**Background:**

Concentric left ventricular hypertrophy (cLVH) is a common left ventricular geometric pattern in patients undergoing maintenance dialysis, including peritoneal dialysis (PD). The relationship between cLVH at PD initiation and the prognosis of patients remains unclear, however. This study aimed to investigate the impact of cLVH at PD initiation on patient survival and major adverse cardiovascular events (MACE).

**Methods:**

The retrospective cohort study included 131 patients who underwent echocardiography during the PD initiation period. Based on echocardiographic measurements, cLVH was defined as a condition with increased LV mass index and increased relative wall thickness. The relationship between cLVH and the prognosis was assessed.

**Results:**

Concentric LVH was identified in 29 patients (22%) at PD initiation, and patient survival, MACE-free survival and PD continuation were significantly reduced in the cLVH group compared with the non-cLVH group. In the Cox regression analysis, cLVH was demonstrated as an independent risk factor of mortality (HR [95%CI]: 3.32 [1.13–9.70]) for all patients. For patients over 65 years old, cLVH was significantly associated with mortality and MACE (HR [95%CI]: 3.51 [1.06–11.58] and 2.97 [1.26–7.01], respectively). Serum albumin at PD initiation was independently correlated with cLVH.

**Conclusions:**

In our study, cLVH at PD initiation was independently associated with survival in all patients and with both survival and MACE in elderly patients. Evaluation of LV geometry at PD initiation might therefore help identify high-risk patients. Further studies involving larger numbers of patients are needed to confirm the findings from this study and clarify whether treatment interventions for factors such as nutrition status could ameliorate cLVH and improve patient outcomes.

**Supplementary Information:**

The online version contains supplementary material available at 10.1186/s12882-021-02321-1.

## Background

Death from cardiovascular diseases (CVDs) is recognized as a significant problem among end-stage kidney disease (ESKD) patients. Cardiovascular disease mortality in ESKD patients is 20 times higher than in the general population and data from the US Renal Data System (2018) showed that CVD accounts for approximately 39% of deaths among patients on dialysis [1]. Left ventricular hypertrophy (LVH) is considered to be one of the risk factors of CVD and death in patients with chronic kidney disease (CKD), including ESKD [2]*.* Left ventricular hypertrophy is also regarded as one of the common cardiovascular complications of dialysis. Among patients treated by hemodialysis (HD) or peritoneal dialysis (PD), the prevalence of LVH is reported to be approximately 75% [3].

In dialysis patients, both traditional risk factors of CVD, such as smoking, hypertension, and diabetes mellitus as well as non-traditional risk factors, such as volume overload, anemia, chronic kidney disease-mineral and bone disorder, uremic toxins, malnutrition, and chronic inflammation can lead to cardiac damage, including LVH [4, 5]. In CKD patients, it was reported that several factors like older age, hypertension, volume overload, anemia, uremia, and hypoalbuminemia were related to the presence of LVH [6].

Left ventricular (LV) geometry can be evaluated noninvasively by echocardiography. Left ventricular geometry is classified into four patterns based on the results of echocardiographic measurements, the left ventricular mass index (LVMI), and the relative wall thickness (RWT): concentric LVH (increased LVMI and RWT); eccentric LVH (increased LVMI but normal RWT); concentric remodeling (normal LVMI but increased RWT); and normal (normal LVMI and RWT) [7].

In the previous report, concentric LVH (cLVH) was the most prevalent LV geometric pattern in dialysis patients, including PD patients [8]. Both hypertension (pressure overload) and fluid excess (volume overload) often contribute to cLVH in ESKD patients. In cLVH, because adaptation to preload decrease is impaired due to LV stiffness, coronary perfusion and systemic circulation can be easily decompensated [9]. For non-dialysis patients, it was reported that the presence of cLVH is related to high mortality and incidence of CVD [10].

LVMI is a widely accepted indicator of patient survival in dialysis patients, and previous reports have shown that LVMI predicts all-cause mortality and cardiovascular death in PD patients [2, 11]. However, to our knowledge, no studies have revealed the relationship between the presence of cLVH among four geometric patterns at PD initiation and mortality or incidence of major adverse cardiovascular events (MACE) during PD therapy. Therefore, we performed a retrospective cohort study to examine the impact of cLVH at PD initiation on patient survival, MACE, and PD technique survival. Furthermore, we investigated clinical factors at PD initiation that may be associated with cLVH.

## Methods

### Patients and data collection

This retrospective cohort study was conducted at a single center (The University of Tokyo Hospital) and approved by the Institutional Review Board of the University of Tokyo (#2879). As this study retrospectively collected the data from medical records, written consent was waived. The participants were 148 adult patients who started PD therapy as their first renal replacement therapy between 2001 and 2018. After excluding subjects who lacked echocardiogram records at PD initiation, however, 131 patients were enrolled. All patients were treated using biocompatible neutral pH solution for PD therapy.

Clinical and laboratory data at PD initiation were collected. These data included age, gender, diabetes, CVD before PD initiation, use of automated PD devices, use of renin-angiotensin system (RAS) inhibitors, use of beta-blockers, use of anti-platelet agents, body mass index, systolic and diastolic blood pressure, daily urine volume, hemoglobin, serum albumin, blood urea nitrogen, serum creatinine, corrected calcium, phosphate, intact PTH (iPTH), C-reactive protein (CRP, logarithmic transformed), total cholesterol, triglyceride, renal weekly Kt/V, total weekly Kt/V, and the ratio of dialysate-to-plasma creatinine concentration at 4 h (D/Pcre) evaluated by peritoneal equilibration test using 2.5% glucose 2 L solution.

### Echocardiographic evaluation

Standard transthoracic echocardiography was performed as part of standard practice at PD initiation. Left ventricular end-diastolic diameter (LVDd) and end-systolic diameter (LVDs), interventricular septum thickness (IVST), and posterior wall thickness (PWT) during diastole were evaluated by two-dimensional echocardiography according to the recommendations of the American Society of Echocardiography (ASE) using commercially available equipment [12]. All echocardiographic examinations had been performed for medical necessity and the measurement data were confirmed by cardiologists. Left ventricular ejection fraction (EF) was calculated using the Teichholz method and left ventricular mass (LVM) and LVMI were calculated using the following equations [12]:

LVM = 0.8 × (1.04 × ([(LVDd) + (PWT) + (IVST)]^3^ –[LVDd]^3^)) + 0.6 g [g].

LVMI = LVM/(body surface area) [g/m^2^].

(Body surface area was calculated using the DuBois equation).

Relative wall thickness was calculated as 2 × PWT/LVDd [12].

In general, cLVH has both increased LVH and elevated RWT when LV geometry is classified into four groups according to LVMI and RWT. In this study, cLVH was defined as follows: LVMI > 115 g/m^2^ for males and > 95 g/m^2^ for females, combined with RWT > 0.42 [12]. LVH was also defined as follows: LVMI > 115 g/m^2^ for males and > 95 g/m^2^ for females.

### Outcomes

The primary outcome of this study was all-cause mortality until six months after PD cessation. The secondary outcome was occurrence of MACE during PD continuation. Major adverse cardiovascular events were defined for this study as follows: coronary artery disease that needs to be treated by angioplasty or coronary artery bypass; stroke; peripheral artery disease requiring hospitalization; and death. Peritoneal dialysis technique failure-free survival (PD technique survival) was also evaluated. PD technique failure was defined as a transfer from PD to HD for more than four weeks due to any cause, such as peritonitis, PD catheter infection, ultrafiltration failure, and insufficient solute clearance.

### Statistical analysis

Continuous data were expressed as mean ± standard deviation or median [interquartile range]. Categorical data were expressed as counts and proportions. Continuous data were compared using Student’s t-test or Mann–Whitney U test. Categorical data were compared using chi-squared test or Fisher’s exact test.

The incidence of death and MACE in the cLVH group compared to the other three LV geometry groups was examined by using the chi-square test and residual analysis.

The differences in patient survival, incidence of MACE, and PD technique survival rate between groups with and without cLVH were compared using the Kaplan–Meier method and the log-rank test.

Cox proportional hazards model analysis was performed to examine whether cLVH at PD initiation was an independent predictor of death or MACE. Candidate variables were selected among parameters that were considered important (e.g., age, CVD before PD initiation, use of RAS inhibitor, pulse pressure, diabetes, log CRP, and iPTH) with caution for multicollinearity. In model 1, the number of variables was two for the analysis of death and was to be less than one-tenth of the number of outcomes for the analysis of MACE. In other models of death and MACE, the number of variables was to be less than one-fifth of the number of outcomes. Cox proportional hazards model analysis including LVMI instead of cLVH was also performed.

Factors associated with cLVH at PD initiation were identified using a binary logistic regression analysis. Candidate variables were selected from parameters which were considered important (age, hemoglobin, log CRP, and albumin). A *p*-value < 0.05 was considered statistically significant and all statistical analyses were conducted using software (JMP 9.0; SAS Institute Inc., Cary, NC).

## Results

### Patient data and characteristics

Baseline characteristics at PD initiation of all 131 patients and of groups with and without cLVH are shown in Table 1. The baseline characteristics at PD initiation of enrolled and excluded patients are shown in Additional Table 1. The baseline characteristics at PD initiation of 51 elderly patients (above 65 years old) are shown in Additional Table 2. Concentric LVH at PD initiation was identified in 29 patients (22%) overall and 18 elderly patients (35%). Age and log CRP were significantly higher, and hemoglobin and serum albumin were significantly lower in the cLVH group compared with the non-cLVH group (Table 1 and Additional Table 2).

**Table 1:** Patient characteristics of groups with and without cLVH at PD initiation in all patients (*N* = 131)

Variables	Total	cLVH(−)	cLVH(+)	*p* value
	(*N* = 131)	(*N* = 102)	(*N* = 29)	
Age (years)	60.8 ± 12.7	58.2 ± 11.8	70.1 ± 11.2	< 0.001
Male gender (*n* [%])	97 [74%]	80 [78%]	17 [59%]	0.032
Diabetes (*n* [%])	44 [34%]	34 [33%]	10 [34%]	0.909
CVD before PD initiation	25 [19%]	16 [16%]	9 [31%]	0.064
(*n* [%])				
Automated PD (*n* [%])	117 [89%]	93 [92%]	24 [83%]	0.198
RAS inhibitor (*n* [%])	111 [85%]	90 [88%]	21 [72%]	0.037
Beta-blocker (n [%])	16 [12%]	11 [11%]	5 [17%]	0.347
Anti-platelet agent (n [%])	39 [30%]	27 [26%]	12 [41%]	0.166
Body mass index (kg/m^2^)	22.2 ± 3.6	22.4 ± 3.4	21.4 ± 3.9	0.177
Systolic blood pressure (mmHg)	132 ± 18	131 ± 18	138 ± 18	0.079
Diastolic blood pressure (mmHg)	77 ± 12	78 ± 12	72 ± 11	0.036
Pulse pressure (mmHg)	56 ± 15	53 ± 13	65 ± 18	< 0.001
Urine volume (ml/day)	1100 [760, 1575]	1200 [800, 1538]	1050 [650, 1600]	0.241
Hemoglobin (g/dl)	11.0 ± 1.1	11.1 ± 1.0	10.4 ± 1.1	0.002
Albumin (g/dl)	3.6 ± 0.4	3.7 ± 0.4	3.2 ± 0.5	< 0.001
Blood urea nitrogen (mg/dl)	52.4 ± 13.2	53.0 ± 12.7	50.5 ± 14.8	0.381
Creatinine (mg/dl)	6.8 ± 2.4	7.2 ± 2.4	5.7 ± 2.1	0.004
Corrected calcium (mg/dl)	9.0 ± 0.7	9.0 ± 0.6	9.1 ± 0.8	0.448
Phosphate (mg/dl)	4.8 ± 1.1	4.8 ± 1.1	4.5 ± 1.1	0.185
iPTH (pg/ml)	207 [137, 362]	211 [149, 381]	150 [79, 215]	0.002
Log CRP	−0.86 ± 0.58	−0.95 ± 0.56	−0.55 ± 0.54	< 0.001
Total cholesterol (mg/dl)	197 ± 41	198 ± 41	196 ± 41	0.824
Triglyceride (mg/dl)	144 [100, 185]	148 [106, 187]	109 [81, 166]	0.039
Renal weekly Kt/V	1.17 ± 0.61	1.19 ± 0.61	1.12 ± 0.61	0.601
Total weekly Kt/V	2.23 ± 0.58	2.24 ± 0.58	2.17 ± 0.57	0.543
D/Pcre	0.58 ± 0.11	0.57 ± 0.10	0.62 ± 0.12	0.022
LVMI (g/m^2^)	99.1 [81.2, 121.6]	91.4 [77.3, 107.4]	135.0 [123.1, 162.6]	< 0.001
RWT	0.43 [0.38, 0.51]	0.40 [0.36, 0.48]	0.51 [0.46, 0.62]	< 0.001

Values are expressed as mean ± standard deviation, median [interquartile range] or number [percentage]

The duration of the observational period was 40.4 [23.6–65.0] months for all patients and 33.6 [18.6–58.8] months for elderly patients. Overall, 16 (12%) patients died, 37 (28%) patients developed MACE, and 78 (60%) patients discontinued PD during the observation period. In the group of patients aged over 65 years, 14 (27%) patients died, 25 (49%) patients developed MACE, and 32 (63%) patients discontinued PD during the observation period.

### Mortality and MACE in patients with cLVH at PD initiation

Distributions of the LV geometry of all patients with information about mortality and MACE incidence in each category are shown in Additional Fig. 1. The chi-square test and residual analysis showed that the cLVH group had significantly higher mortality and MACE incidence than the other three groups (*p* < 0.001 and *p* = 0.009, respectively, as shown in Additional Table 3), however, the eccentric LVH group did not.

**Fig. 1:**
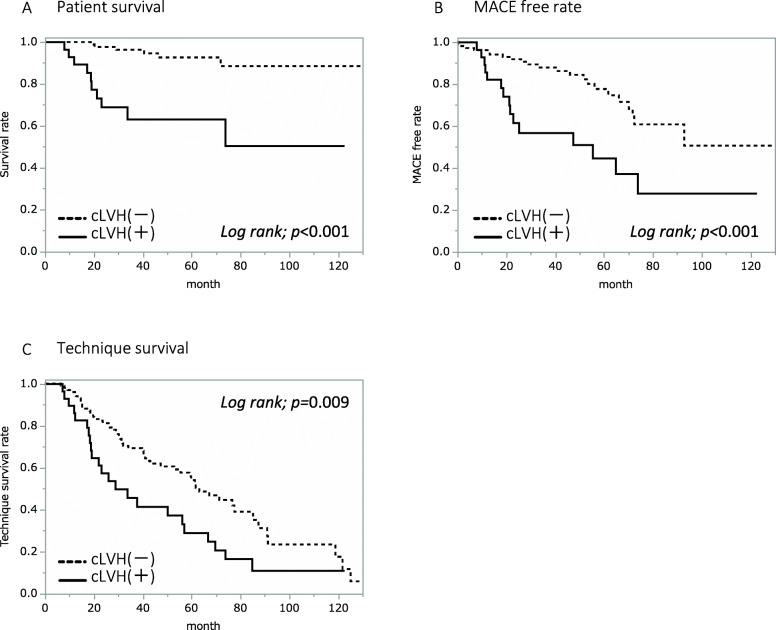
Survival, MACE-free rate, and PD technique survival rate in all patients**.** Comparison of survival (**a**), MACE-free rate (**b**), and PD technique survival rate (**c**) between groups with (*N* = 29) and without (*N* = 102) cLVH in 131 patients by Kaplan–Meier plots and a log-rank test

Concentric LVH was associated with higher incidence of death and MACE (odds ratio [95%CI]: 8.42 [2.73–25.95] and 3.90 [1.64–9.28], respectively) compared with non-cLVH (Additional Table 4). Similar results were observed for LVH (odds ratio [95%CI]: 5.71 [1.84–17.72] for death and 2.61 [1.19–5.76] for MACE) (Additional Table 4).

**Fig. 2:**
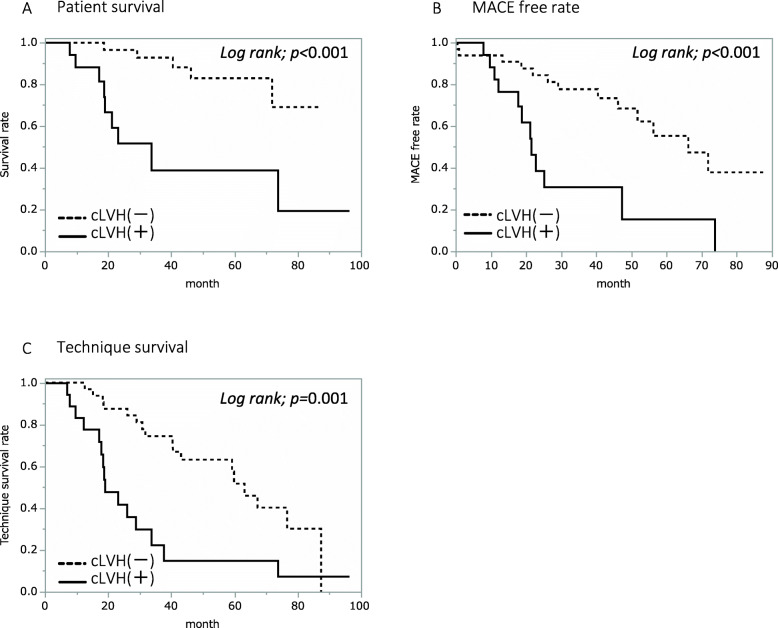
Survival, MACE-free rate, and PD technique survival rate in elderly patients**.** Comparison of survival (**a**), MACE-free rate (**b**), and PD technique survival rate (**c**) between groups with (*N* = 18) and without (*N* = 33) cLVH in 51 elderly patients by Kaplan–Meier plots and a log-rank test

When all patients were divided into two groups, being one with and the other without cLVH, Kaplan-Meier analysis revealed that patient survival (*p* < 0.001), MACE-free rate (*p* < 0.001), and PD technique survival (*p* = 0.009) were all significantly lower in the cLVH group (see Fig. 1). The analyses of the elderly patients had similar results with the cLVH group showing a significantly lower survival (*p* < 0.001), lower MACE-free rate (*p* < 0.001), and lower PD technique survival (*p* = 0.001) compared with the non-cLVH group (see Fig. 2).

### Impact of cLVH at PD initiation on the prognosis of PD patients

The Cox proportional hazards model for all patients revealed that cLVH was independently associated with death (HR [95%CI]: 3.32 [1.13–9.70] for model 1) after adjusting for other factors (see Table 2 and Additional Table 6). The Cox proportional hazards model for patients aged over 65 years showed that cLVH was independently associated with both death (HR [95%CI]: 3.51 [1.06–11.58] for model 1) and MACE (HR [95%CI]: 2.97 [1.26–7.01] for model 1) after adjusting for other factors (see Table 2 and Additional Table 6). LVMI was also independently associated with death and MACE (Additional Table 5).

**Table 2:** Cox regression hazard models on death and MACE for all or aged over 65 patients

	All patients						Patients aged over 65				
**Death**		Univariate		Multivariate		**Death**		Univariate		Multivariate	
	Variable	Hazard ratio [95%CI]	*p* value	Hazard ratio [95%CI]	*p* value		Variable	Hazard ratio [95%CI]	*p* value	Hazard ratio [95%CI]	*p* value
Model 1	Age (per 1 year)	1.17	< 0.001	1.15	< 0.001	Model 1	Age (per 1 year)	1.23	< 0.001	1.20	0.001
		[1.10–1.26]		[1.09–1.24]				[1.12–1.38]		[1.08–1.35]	
	cLVH (+ vs. −)	7.35	< 0.001	3.32	0.029		cLVH (+ vs. −)	6.30	0.001	3.51	0.039
		[2.67–20.30]		[1.13–9.70]				[2.04–19.45]		[1.06–11.58]	
Model 2	Age (per 1 year)	1.17	< 0.001	1.15	< 0.001	Model 2	Age (per 1 year)	1.23	< 0.001	1.21	0.001
		[1.10–1.26]		[1.08–1.23]				[1.12–1.38]		[1.08–1.36]	
	RAS inhibitor (− vs. +)	5.09	0.001	1.86	0.229		RAS inhibitor (− vs. +)	2.35	0.121	1.53	0.444
		[1.89–13.70]		[0.68–5.13]				[0.80–6.90]		[0.52–4.53]	
	cLVH (+ vs. −)	7.35	< 0.001	3.22	0.031		cLVH (+ vs. −)	6.30	0.001	3.43	0.039
		[2.67–20.30]		[1.11–9.30]				[2.04–19.45]		[1.06–11.04]	
Model 3	Age (per 1 year)	1.17	< 0.001	1.15	< 0.001	Model 3	Age (per 1 year)	1.23	< 0.001	1.19	0.001
		[1.10–1.26]		[1.08–1.23]				[1.12–1.38]		[1.07–1.36]	
	Log CRP (per 0.523)	1.94	0.026	1.22	0.661		log CRP (per 0.523)	2.38	0.025	1.25	0.711
		[1.00–3.31]		[0.46–2.73]				[1.05–4.97]		[0.36–3.84]	
	cLVH (+ vs. −)	7.35	< 0.001	3.16	0.040		cLVH (+ vs. −)	6.30	0.001	3.38	0.046
		[2.67–20.30]		[1.07–9.98]				[2.04–19.45]		[1.00–11.40]	
Model 4	Age (per 1 year)	1.17	< 0.001	1.15	< 0.001	Model 4	Age (per 1 year)	1.23	< 0.001	1.20	0.001
		[1.10–1.26]		[1.08–1.24]				[1.12–1.38]		[1.08–1.35]	
	CVD before PD initiation (+ vs. −)	2.22	0.141	1.21	0.732		CVD before PD initiation (+ vs. −)	1.05	0.931	1.06	0.921
		[0.77–6.40]		[0.41–3.57]				[0.32–3.42]		[0.32–3.53]	
	cLVH (+ vs. −)	7.35	< 0.001	3.33	0.028		cLVH (+ vs. −)	6.30	0.001	3.52	0.039
		[2.67–20.30]		[1.14–9.73]				[2.04–19.45]		[1.07–11.63]	
Model 5	Pulse pressure	1.06	< 0.001	1.03	0.052	Model 5	Pulse pressure	1.04	0.011	1.01	0.661
		[1.03–1.10]		[1.00–1.07]				[1.01–1.07]		[0.97–1.05]	
	RAS inhibitor (− vs. +)	5.09	0.001	2.48	0.109		RAS inhibitor (− vs. +)	2.35	0.121	1.37	0.620
		[1.89–13.70]		[0.82–7.53]				[0.80–6.90]		[0.39–4.75]	
	cLVH (+ vs. −)	7.35	< 0.001	3.23	0.066		cLVH (+ vs. −)	6.30	0.001	4.68	0.028
		[2.67–20.30]		[0.92–11.30]				[2.04–19.45]		[1.18–18.56]	
**MACE**		Univariate		Multivariate		**MACE**		Univariate		Multivariate	
	Variable	Hazard ratio [95%CI]	*p* value	Hazard ratio [95%CI]	*p* value		Variable	Hazard ratio [95%CI]	*p* value	Hazard ratio [95%CI]	*p* value
Model 1	Age (per 1 year)	1.09	< 0.001	1.07	< 0.001	Model 1	Age (per 1 year)	1.10	0.008	1.07	0.059
		[1.05–1.12]		[1.04–1.11]				[1.02–1.18]		[1.00–1.15]	
	CVD before PD initiation (+ vs. −)	3.48	< 0.001	2.14	0.038		cLVH (+ vs. −)	3.69	0.002	2.97	0.013
		[1.75–6.91]		[1.04–4.38]				[1.63–8.35]		[1.26–7.01]	
	cLVH (+ vs. −)	3.06	< 0.001	2.13	0.032						
		[1.58–5.92]		[1.07–4.25]							
Model 2	Age (per 1 year)	1.09	< 0.001	1.08	< 0.001	Model 2	Age (per 1 year)	1.10	0.008	1.05	0.174
		[1.05–1.12]		[1.04–1.12]				[1.02–1.18]		[0.98–1.14]	
	CVD before PD initiation (+ vs. −)	3.48	< 0.001	2.31	0.026		CVD before PD initiation (+ vs. −)	1.77	0.192	1.35	0.527
		[1.75–6.91]		[1.11–4.84]				[0.77–4.05]		[0.53–3.42]	
	Diabetes	1.85	0.066	2.05	0.047		log CRP (per 0.523)	2.22	0.007	1.53	0.273
	(+ vs. −)	[0.96–3.55]		[1.01–4.17]				[1.20–3.90]		[0.67–3.11]	
	RAS inhibitor (− vs. +)	1.79	0.147	0.70	0.431		cLVH (+ vs. −)	3.69	0.002	2.81	0.025
		[0.81–3.92]		[0.29–1.69]				[1.63–8.35]		[1.12–6.92]	
	log CRP (per 0.523)	1.33	0.221	1.03	0.932						
		[0.80–2.02]		[0.58–1.82]							
	cLVH (+ vs. −)	3.06	< 0.001	1.79	0.138						
		[1.58–5.92]		[0.83–3.87]							

### Clinical parameters correlated with cLVH at PD initiation

In the binary logistic regression analysis for all patients, a statistically significant association was found between the presence of cLVH and age and cLVH and serum albumin (see Table 3). In analysis of the subgroup of patients aged over 65 years, only serum albumin was found to have a significant association with cLVH (see Table 3).

**Table 3:** Binary logistic regression analysis for the presence of cLVH

All patients					
	Univariate		Multivariate		
Variable	Odds ratio [95%CI]	*p* value	β [95%CI]	Odds ratio [95%CI]	*p* value
Age (per 1 year)	1.09 [1.05–1.14]	< 0.001	0.06 [0.01–0.11]	1.06 [1.01–1.11]	0.017
Hemoglobin (per 1 g/dl)	0.51 [0.32–0.79]	0.002	−0.51 [−1.10–0.00]	0.60 [0.34–1.00]	0.062
log CRP (per 1 unit)	3.55 [1.61–7.81]	< 0.001	0.24 [−0.80–1.25]	1.28 [0.47–3.49]	0.630
Albumin (per 1 g/dl)	0.09 [0.03–0.28]	< 0.001	−1.42 [−2.90– − 0.08]	0.24 [0.06–0.93]	0.044
**Patients aged over 65 years**					
	**Univariate**		**Multivariate**		
Variable	Odds ratio [95%CI]	*p* value	β [95%CI]	Odds ratio [95%CI]	*p* value
Age (per 1 year)	1.16 [1.04–1.29]	0.003	0.11 [−0.01–0.22]	1.11 [0.99–1.25]	0.052
Albumin (per 1 g/dl)	0.09 [0.02–0.50]	0.002	−1.86 [−3.64– −0.09]	0.15 [0.03–0.92]	0.027

## Discussion

This study investigated whether cLVH at PD initiation was associated with prognosis in PD patients. As it is known that LVH is strongly affected by aging in general, we also analyzed data from patients aged over 65 years. Our results showed that the cLVH group had a significantly higher risk of death, MACE, and PD discontinuation compared with the non-cLVH group for all patients and elderly patients. Cox analyses revealed that cLVH at PD initiation was independently associated with death in all patients and associated with both death and MACE in elderly patients. Serum albumin at PD initiation was negatively associated with the presence of cLVH.

Previous studies have shown that the complications of LVH and the value of LVMI are independent risk factors of mortality and CVD in maintaining dialysis patients [3, 7]. Indeed, LVMI at PD initiation was independently associated with mortality and MACE in this study (Additional Table 5). There have been few studies that have investigated the relationship between LV geometry and prognosis in PD patients, however. To our knowledge, this is the first report that focuses on the relationship between LV geometry at PD initiation and the prognoses of adult patients. We demonstrated that cLVH at PD initiation was an independent predictor of poor prognoses, i.e. mortality and cardiovascular events.

The prevalence of LVH have been reported to range from 44 to 58% ([13, 14]) among maintenance PD patients. In our study, the prevalence of LVH was 33%, lower than previous reports. The reasons for the difference in these rates are obscure, however, regarding the fact that residual renal function (RRF) is reported to have an impact on LVH [15], one reason is presumably the shorter PD duration until the time of echocardiography. In our cohort, echocardiography was performed at 2.8 ± 1.2 months from the first dialysis.

There are a few reports on the features of LV geometry in ESKD patients, and the prevalence of cLVH varies from report to report. Although 22% of PD patients in our study had cLVH, a previous report showed that only 11.5% of PD patients had cLVH [16]. One of the reasons for such a large difference in cLVH prevalence may be aging. In general, older age is strongly correlated with LVH [17, 18]. Although the mean age of patients was 42 years in the previous study that showed a lower prevalence of cLVH, it was 60.8 years in our study [16]. In another study including both HD (66%) and PD (34%) patients, in which the mean age was 51.9 years, cLVH was identified in almost half of all patients (49%) and was the most frequent LV geometry model [8].

The degree of fluid status and/or hypertension before and after starting dialysis may also affect the prevalence of cLVH. As the LVs of dialysis patients are often exposed to pressure overload (due to hypertension) and volume overload (due to excess fluid), LVH in ESKD patients tends to have the features of both concentric and eccentric hypertrophy [19]. The management of the traditional and non-traditional risk factors of CVD can also affect LVH in dialysis patients [4, 5].

Cox analyses showed that age was an independent predictor of death and MACE for all patients (Table 2). Age was significantly associated with cLVH at PD initiation as well as serum albumin in all patients (Table 3). The associations were not observed in elderly patients. Previous reports have shown that age is independently associated with LV concentric change [17, 18] and recent basic studies have reported that several cellular and molecular changes, such as reduction of cardiomyocytes, replacement hypertrophy of residual cardiomyocytes, deposition of extracellular matrix, and extracellular fibrosis were observed in LV with aging [20, 21]. These histological changes may lead LVs to undergo undesirable morphological changes, such as LVH.

Although not all the mechanisms of the “aging of cardiomyocytes” are elucidated, the following factors have been shown to play crucial roles: oxidative stress; inflammation; reduced cellular protection and repair; telomere attrition; abnormal cellular metabolism; post-translational modifications; and altered gene expression [22, 23].

From the results of the Cox hazards model, it was found that cLVH was an independent risk factor for death in all patients and for both death and MACE in the elderly patients (Table 2). Possible pathophysiological mechanisms by which cLVH affects the prognosis of PD patients need to be clarified as cLVH often occurs under pressure overload. One of the mechanisms of cLVH exacerbation is considered to be the result of stiffer myocardial remodeling of the LV, which leads to a higher risk of CVD [24]. On the other hand, eccentric LVH is more likely due to chronic volume overload [24, 25]. As shown in Additional Table 3, the mortality and MACE incidence in the cLVH group were significantly higher, however, not in the eccentric LVH group. These results might implicate the importance of assessing not only the LVMI value but also the RWT, which seems to reflect myocardial remodeling more sensitively.

Our study demonstrates that serum albumin levels at PD initiation are associated with the existence of cLVH. Previous reports have shown a strong association between low serum albumin levels and LV dilation in CKD and ESKD patients, including PD [26, 27]. This relationship may indicate that malnutrition and inflammation could have a deleterious influence on cardiac structure and function. In fact, cardiac atrophy, hypertrophy, fibrosis, and low cardiac output were observed in children with forms of malnutrition, such as kwashiorkor and marasmus [28].

An increase of inflammation markers such as plasma hs-CRP and IL-6 is also reported to be associated with LVH and cardiac systolic dysfunction [26]. Considering that changes in LV geometry will be induced over a long period, our results suggest that amelioration of nutritional status until PD initiation may prevent undesirable LV structural changes like cLVH and could decrease the risk of CVD and death after starting PD. Further studies are needed to investigate whether the improvement and maintenance of nutritional condition after PD initiation could ameliorate cLVH and improve prognoses.

There are several limitations in our study. First, our study was single-institution-based, and the sample sizes and number of events are small. For this reason, we cannot conclude that LV geometry must be assessed in every patient at PD induction. Further research, including more patients from multiple centers, will be needed to clarify the significance of cLVH on the prognosis of PD patients.

Second, most of the patients were treated using automated PD. This population bias may make it difficult to apply the results to all patients, especially those treated with CAPD. Further multi-center studies including larger numbers of both CAPD and automated PD patients are needed.

Third, we obtained echocardiographic data only at PD initiation and did not evaluate changes of LV geometry over time before and after PD initiation. Although changes in LV geometry over time might provide more accurate predictions regarding patient prognoses, our results showed that the long-term prognoses of PD patients could be predicted by LV geometry at an early point of PD initiation. Considering that morphologic changes in the LV need time and are affected by complicated factors such as fluid and hypertension, cLVH at PD initiation could be regarded as the result of CKD management before ESKD.

A recent report showed that reduced estimated glomerular filtration rate is associated with cardiac hypertrophy and fibrosis in CKD patients, especially those with anemia [29]. In the future, the clinical significance of changes in LV geometry over time should be clarified and modifiable factors associated with changes in cLVH should be identified to establish treatment strategies in PD patients.

## Conclusions

In conclusion, our study suggests that the presence of cLVH at PD initiation may be a predictor of prognoses, and serum albumin at PD initiation was negatively correlated with the presence of cLVH. Evaluation of cLVH at PD initiation might be useful for the stratification of PD patients with a high risk of CVD and mortality. To clarify the impact of cLVH at PD initiation on prognoses, additional research with larger numbers of patients are required. Further studies are also needed to reveal whether changes in cLVH during PD could have clinical significance and whether nutrition management could improve prognoses by ameliorating cLVH in PD patients.

## Supplementary Information

**Additional file 1: Table S1**. Patient characteristics of groups included in and excluded from this study (*N* = 148). Values are expressed as mean ± standard deviation, median [interquartile range] or number [percentage].

**Additional file 2: Table S2**. Patient characteristics of groups with and without cLVH at PD initiation in elderly patients (*N* = 51). Values are expressed as mean ± standard deviation, median [interquartile range] or number [percentage].

**Additional file 3: Table S3**. The prevalence of death and MACE in each LV geometry category (*N* = 131). The results of chi-square test and residual analysis are shown.

**Additional file 4: Table S4**. Two-by-two contingency tables and odds ratios based on LV shapes (cLVH or LVH) and outcomes (death or MACE) (*N* = 131). Concentric LVH was defined as follows: LVMI > 115 g/m^2^ for males and > 95 g/m^2^ for females, combined with RWT > 0.42. LVH was defined as follows: LVMI > 115 g/m^2^ for males and > 95 g/m^2^ for females.

**Additional file 5 Table S5**. Cox regression hazard models on death and MACE for all patients and for patients aged over 65 (adjusted for variables including LVMI).

**Additional file 6 Table S6**. Cox regression hazard models on death and MACE for all patients and for patients aged over 65 (adjusted for variables including iPTH).

**Additional file 7 Fig. S1** Distributions of left ventricular mass index (LVMI) and relative wall thickness (RWT) in 131 patients are shown with information about mortality (A) and MACE incidence (B) in each category of LV geometry. Males and females are indicated as squares and circles, respectively. Patients with death (A) and MACE (B) are indicated by closed shapes. Mortality (A) and MACE incidence (B) in each category are shown.

## Data Availability

The datasets used and analysed during the current study are available from the corresponding author on reasonable request.

## Abbreviations

ASE: American Society of Echocardiography
CKD: Chronic kidney disease
cLVH: Concentric left ventricular hypertrophy
CRP: C-reactive protein
CVD: Cardiovascular disease
D/Pcre: The ratio of dialysate-to-plasma creatinine concentration at 4 h
EF: Ejection fraction
ESKD: End-stage kidney disease
HD: Hemodialysis
IVST: Interventricular septum thickness
iPTH: Intact PTH
LV: Left ventricular, left ventricle
LVDd: Left ventricular end-diastolic diameter
LVDs: Left ventricular end-systolic diameter
LVH: Left ventricular hypertrophy
LVM: Left ventricular mass
LVMI: Left ventricular mass index
MACE: Major adverse cardiovascular events
PD: Peritoneal dialysis
PWT: Posterior wall thickness
RAS: Renin-angiotensin system
RWT: Relative wall thickness
