# Supplementary material for: Prognostic significance of concentric left ventricular hypertrophy at peritoneal dialysis initiation
Source: BMC Nephrol. 2021 Apr 16;22:135. doi: 10.1186/s12882-021-02321-1 (PMC8052641; doi:10.1186/s12882-021-02321-1)
Supplement: Supplementary file 1 — Additional file 1: Table S1. Patient characteristics of groups included in and excluded from this study (N = 148). Values are expressed as mean ± standard deviation, median [interquartile range] or number [percentage]. [file 12882_2021_2321_MOESM1_ESM.pdf]

Variables	Included (N=131)	Excluded (N=17)	*p* value
Age (years)	60.8 ± 12.7	66.6 ± 18.9	0.107
Male gender (*n* [%])	97 [74%]	14 [82%]	0.460
Diabetes (*n* [%])	44 [34%]	4 [24%]	0.408
CVD before PD initiation (*n* [%])	25 [19%]	7 [41%]	0.038
Automated PD (*n* [%])	117 [89%]	14 [82%]	0.401
RAS inhibitor (*n* [%])	111 [85%]	12 [69%]	0.110
Beta-blocker (n [%])	16 [12%]	5 [29%]	0.055
Anti-platelet agent (n [%])	39 [30%]	7 [41%]	0.265
Body mass index (kg/m^2)	22.2 ± 3.6	22.6 ± 3.4	0.698
Systolic blood pressure (mmHg)	132 ± 18	137 ± 20	0.372
Diastolic blood pressure (mmHg)	77 ± 12	72 ± 15	0.147
Pulse pressure (mmHg)	56 ± 15	65 ± 17	0.026
Urine volume (ml/day)	1100 [760, 1575]	1150 [888, 1440]	0.948
Hemoglobin (g/dl)	11.0 ± 1.1	9.9 ± 1.1	<0.001
Albumin (g/dl)	3.6 ± 0.4	3.3 ± 0.5	0.037
Blood urea nitrogen (mg/dl)	52.4 ± 13.2	49.3 ± 15.4	0.381
Creatinine (mg/dl)	6.8 ± 2.4	5.4 ± 2.0	0.019
Corrected calcium (mg/dl)	9.0 ± 0.7	8.6 ± 0.6	0.007
Phosphate (mg/dl)	4.8 ± 1.1	4.1 ± 0.8	0.016
iPTH (pg/ml)	207 [137, 362]	350 [213, 400]	0.064
Log CRP	−0.86 ± 0.58	−0.34 ± 0.31	<0.001
Total cholesterol (mg/dl)	197 ± 41	192 ± 32	0.632
Triglyceride (mg/dl)	144 [100, 185]	132 [97, 158]	0.392
Renal weekly Kt/V	1.17 ± 0.61	1.19 ± 0.55	0.952
Total weekly Kt/V	2.23 ± 0.58	2.27 ± 0.58	0.784
D/Pcre	0.58 ± 0.11	0.64 ± 0.14	0.060
Death (%)	16 [12%]	4 [24%]	0.202
MACE (%)	37 [28%]	7 [41%]	0.276

Values are expressed as mean ± standard deviation, median [interquartile range] or number [percentage].
