# Supplementary material for: Prognostic significance of concentric left ventricular hypertrophy at peritoneal dialysis initiation
Source: BMC Nephrol. 2021 Apr 16;22:135. doi: 10.1186/s12882-021-02321-1 (PMC8052641; doi:10.1186/s12882-021-02321-1)
Supplement: Supplementary file 2 — Additional file 2: Table S2. Patient characteristics of groups with and without cLVH at PD initiation in elderly patients (N = 51). Values are expressed as mean ± standard deviation, median [interquartile range] or number [percentage]. [file 12882_2021_2321_MOESM2_ESM.pdf]

Variables	Total (N=51)	cLVH(-) (N=33)	cLVH(+) (N=18)	*p* value
Age (years)	73.7 ± 6.5	71.8 ± 5.3	77.2 ± 7.1	0.003
Male gender (*n* [%])	38 [74%]	26 [79%]	12 [67%]	0.352
Diabetes (*n* [%])	16 [31%]	11 [33%]	5 [28%]	0.690
CVD before PD initiation (n [%])	15 [29%]	9 [27%]	6 [33%]	0.658
Automated PD (*n* [%])	40 [78%]	27 [82%]	13 [72%]	0.436
RAS inhibitor (*n* [%])	40 [78%]	28 [85%]	12 [67%]	0.137
Beta-blocker (n [%])	9 [18%]	6 [18%]	3 [17%]	1.000
Anti-platelet agent (n [%])	14 [27%]	8 [24%]	6 [33%]	0.525
Body mass index (kg/m^2)	22.2 ± 3.6	22.6 ± 3.3	21.4 ± 4.0	0.255
Systolic blood pressure (mmHg)	137 ± 18	134 ± 17	143 ± 18	0.111
Diastolic blood pressure (mmHg)	75 ± 12	75 ± 13	75 ± 11	0.914
Pulse pressure (mmHg)	62 ± 15	59 ± 12	68 ± 17	0.037
Urine volume (ml/day)	1100 [725, 1600]	1100 [800, 1600]	1065 [613, 1505]	0.229
Hemoglobin (g/dl)	10.9 ± 1.1	11.2 ± 0.9	10.4 ± 1.3	0.015
Albumin (g/dl)	3.4 ± 0.4	3.5 ± 0.4	3.1 ± 0.5	0.002
Blood urea nitrogen (mg/dl)	52.7 ± 12.8	51.9 ± 12.2	54.0 ± 14.3	0.596
Creatinine (mg/dl)	6.2 ± 1.8	6.4 ± 1.8	5.9 ± 1.8	0.312
Corrected calcium (mg/dl)	9.1 ± 0.6	9.1 ± 0.6	9.0 ± 0.7	0.805
Phosphate (mg/dl)	4.7 ± 1.1	4.7 ± 1.1	4.7 ± 1.1	0.967
iPTH (pg/ml)	204 [98, 312]	207 [103, 336]	166 [90, 262]	0.245
Log CRP	−0.68 ± 0.55	−0.80 ± 0.51	−0.47 ± 0.56	0.039
Total cholesterol (mg/dl)	194 ± 42	192 ± 40	197 ± 45	0.663
Triglyceride (mg/dl)	145 [104, 174]	160 [113, 175]	117 [72, 160]	0.060
Renal weekly Kt/V	1.14 ± 0.50	1.19 ± 0.53	1.04 ± 0.45	0.299
Total weekly Kt/V	2.14 ± 0.52	2.16 ± 0.51	2.11 ± 0.55	0.742
D/Pcre	0.61 ± 0.11	0.59 ± 0.09	0.65 ± 0.13	0.061
LVMI (g/m^2)	105.4 [91.1, 128.4]	93.7 [82.4, 105.4]	133.9 [122.7, 165.7]	<0.001
RWT	0.45 [0.40, 0.57]	0.41 [0.38, 0.49]	0.58 [0.50, 0.64]	<0.001

Values are expressed as mean ± standard deviation, median [interquartile range] or number [percentage].
