# Supplementary material for: Prognostic significance of concentric left ventricular hypertrophy at peritoneal dialysis initiation
Source: BMC Nephrol. 2021 Apr 16;22:135. doi: 10.1186/s12882-021-02321-1 (PMC8052641; doi:10.1186/s12882-021-02321-1)
Supplement: Supplementary file 3 — Additional file 3: Table S3. The prevalence of death and MACE in each LV geometry category (N = 131). The results of chi-square test and residual analysis are shown. [file 12882_2021_2321_MOESM3_ESM.pdf]

	**Normal**	SD	**Concentric remodeling**	SD	**Concentric LVH**	SD	**Eccentric LVH**	SD	*p* value
**Death**	1/44 (2.3%)	−4.37	4/44 (9.1%)	−1.37	10/29 (34.5%) *	6.46	1/14 (7.1%)	−0.71	<0.001
**MACE**	7/44 (15.9%)	−5.43	12/44 (27.3%)	-0.43	15/29 (51.7%) *	6.81	3/14 (21.4%)	−0.95	0.009

* $p < 0.01$
