# Supplementary material for: Prognostic significance of concentric left ventricular hypertrophy at peritoneal dialysis initiation
Source: BMC Nephrol. 2021 Apr 16;22:135. doi: 10.1186/s12882-021-02321-1 (PMC8052641; doi:10.1186/s12882-021-02321-1)
Supplement: Supplementary file 4 — Additional file 4: Table S4. Two-by-two contingency tables and odds ratios based on LV shapes (cLVH or LVH) and outcomes (death or MACE) (N = 131). Concentric LVH was defined as follows: LVMI > 115 g/m2 for males and > 95 g/m2 for females, combined with RWT > 0.42. LVH was defined as follows: LVMI > 115 g/m2 for males and > 95 g/m2 for females. [file 12882_2021_2321_MOESM4_ESM.pdf]

	death(−)	death(+)	total	Odds Ratio	p value
cLVH(−)	96	6	102		
cLVH(+)	19	10	29	8.42 (2.73–25.95)	<0.001
	MACE(−)	MACE(+)	total	Odds Ratio	p value
cLVH(−)	80	22	102		
cLVH(+)	14	15	29	3.90 (1.64–9.28)	0.002
	death(−)	death(+)	total	Odds Ratio	p value
LVH(−)	83	5	88		
LVH(+)	32	11	43	5.71 (1.84–17.72)	0.003
	MACE(−)	MACE(+)	total	Odds Ratio	p value
LVH(−)	69	19	88		
LVH(+)	25	18	43	2.61 (1.19–5.76)	0.023

cLVH was defined as follows: LVMI >115 g/m^2 for males and >95 g/m^2 for females, combined with RWT > 0.42.

LVH was defined as follows: LVMI >115 g/m^2 for males and >95 g/m^2 for females.
