# Supplementary material for: Prognostic significance of concentric left ventricular hypertrophy at peritoneal dialysis initiation
Source: BMC Nephrol. 2021 Apr 16;22:135. doi: 10.1186/s12882-021-02321-1 (PMC8052641; doi:10.1186/s12882-021-02321-1)
Supplement: Supplementary file 5 — Additional file 5 Table S5. Cox regression hazard models on death and MACE for all patients and for patients aged over 65 (adjusted for variables including LVMI). [file 12882_2021_2321_MOESM5_ESM.pdf]

		All patients					Patients aged over 65			
**Death**		Univariate		Multivariate		**Death**		Univariate		Multivariate	
	Variable	Hazard ratio [95%CI]	*p* value	Hazard ratio [95%CI]	*p* value		Variable	Hazard ratio [95%CI]	*p* value	Hazard ratio [95%CI]	*p* value
Model 1	Age (per 1 year)	1.17 [1.10–1.26]	<0.001	1.16 [1.09–1.25]	<0.001	Model 1	Age (per 1 year)	1.23 [1.12–1.38]	<0.001	1.23 [1.10–1.42]	0.001
	LVMI (per 1 g/m^2)	1.03 [1.02–1.04]	<0.001	1.02 [1.01–1.04]	0.001		LVMI (per 1 g/m^2)	1.03 [1.01–1.05]	<0.001	1.02 [1.01–1.04]	0.003
**MACE**		Univariate		Multivariate		**MACE**		Univariate		Multivariate	
	Variable	Hazard ratio [95%CI]	*p* value	Hazard ratio [95%CI]	*p* value		Variable	Hazard ratio [95%CI]	*p* value	Hazard ratio [95%CI]	*p* value
Model 1	Age (per 1 year)	1.09 [1.05–1.12]	<0.001	1.07 [1.04–1.11]	<0.001	Model 1	Age (per 1 year)	1.10 [1.02–1.18]	0.008	1.08 [1.00–1.17]	0.036
	CVD before PD initiation (+ vs. −)	3.48 [1.75–6.91]	<0.001	2.15 [1.06–4.38]	0.035		LVMI (per 1 g/m^2)	1.02 [1.01–1.03]	0.001	1.02 [1.01–1.03]	0.003
	LVMI (per 1 g/m^2)	1.02 [1.01–1.03]	<0.001	1.02 [1.01–1.02]	0.001						
