# Supplementary material for: Prognostic significance of concentric left ventricular hypertrophy at peritoneal dialysis initiation
Source: BMC Nephrol. 2021 Apr 16;22:135. doi: 10.1186/s12882-021-02321-1 (PMC8052641; doi:10.1186/s12882-021-02321-1)
Supplement: Supplementary file 6 — Additional file 6 Table S6. Cox regression hazard models on death and MACE for all patients and for patients aged over 65 (adjusted for variables including iPTH). [file 12882_2021_2321_MOESM6_ESM.pdf]

	All patients					Patients aged over 65					
**Death**		Univariate		Multivariate		**Death**		Univariate		Multivariate	
	Variable	Hazard ratio [95%CI]	*p* value	Hazard ratio [95%CI]	*p* value		Variable	Hazard ratio [95%CI]	*p* value	Hazard ratio [95%CI]	*p* value
Model 6	Age (per 1 year)	1.17 [1.10–1.26]	<0.001	1.15 [1.09–1.24]	<0.001	Model 6	Age (per 1 year)	1.23 [1.12–1.38]	<0.001	1.21 [1.09–1.37]	0.001
	iPTH (per 1 pg/ml)	1.00 [1.00–1.00]	0.205	1.00 [1.00–1.00]	0.648		iPTH (per 1 pg/ml)	1.00 [0.99–1.00]	0.569	1.00 [0.99–1.00]	0.505
	cLVH (+ vs. −)	7.35 [2.67–20.30]	<0.001	3.24 [1.11–9.42]	0.031		cLVH (+ vs. −)	6.30 [2.04–19.45]	0.001	3.45 [1.07–11.15]	0.038
**MACE**		Univariate		Multivariate		**MACE**		Univariate		Multivariate	
	Variable	Hazard ratio [95%CI]	*p* value	Hazard ratio [95%CI]	*p* value		Variable	Hazard ratio [95%CI]	*p* value	Hazard ratio [95%CI]	*p* value
Model 3	Age (per 1 year)	1.09 [1.05–1.12]	<0.001	1.07 [1.04–1.11]	<0.001	Model 3	Age (per 1 year)	1.10 [1.02–1.18]	0.008	1.05 [0.97–1.13]	0.199
	CVD before PD initiation (+ vs. −)	3.48 [1.75–6.91]	<0.001	2.24 [1.02–4.89]	0.044		CVD before PD initiation (+ vs. −)	1.77 [0.77–4.05]	0.192	1.47 [0.52–4.12]	0.464
	log CRP (per 0.523)	1.33 [0.80–2.02]	0.221	1.01 [0.58–1.76]	0.969		log CRP (per 0.523)	2.22 [1.20–3.90]	0.007	1.55 [0.68–3.18]	0.263
	iPTH (per 1 pg/ml)	1.00 [1.00–1.00]	0.081	1.00 [1.00–1.00]	0.745		iPTH (per 1 pg/ml)	1.00 [1.00–1.00]	0.526	1.00 [1.00–1.00]	0.694
	cLVH (+ vs. −)	3.06 [1.58–5.92]	<0.001	2.18 [1.04–4.54]	0.038		cLVH (+ vs. −)	3.69 [1.63–8.35]	0.002	2.92 [1.14–7.43]	0.025
