# Supplementary figures and images for: Prognostic significance of concentric left ventricular hypertrophy at peritoneal dialysis initiation
Source: BMC Nephrol. 2021 Apr 16;22:135. doi: 10.1186/s12882-021-02321-1 (PMC8052641; doi:10.1186/s12882-021-02321-1)

A

Death

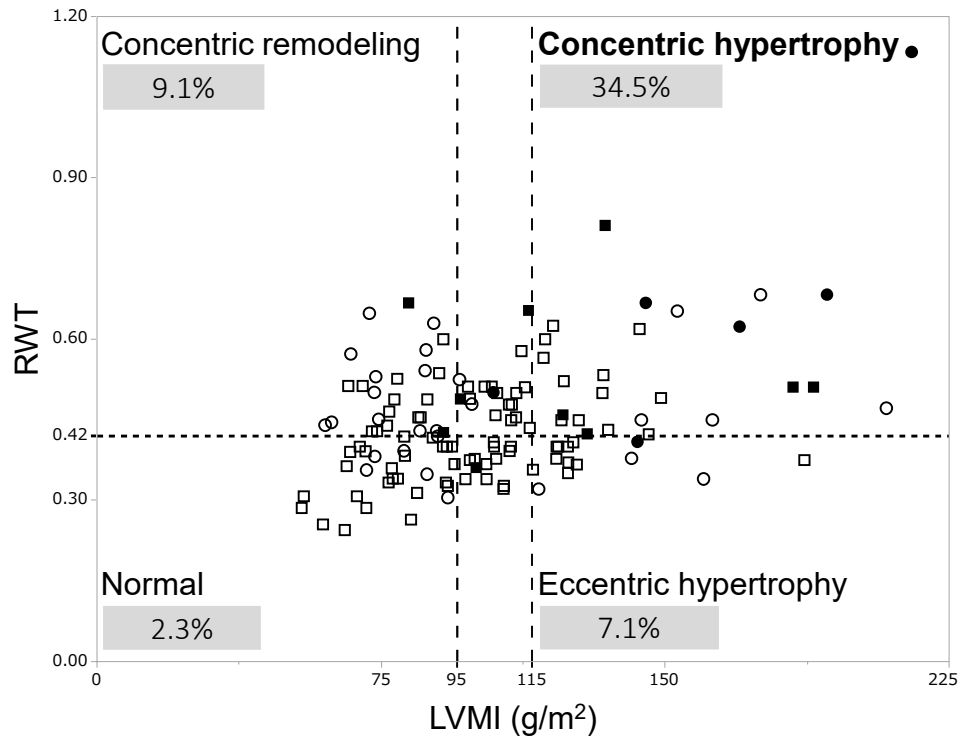

B

MACE

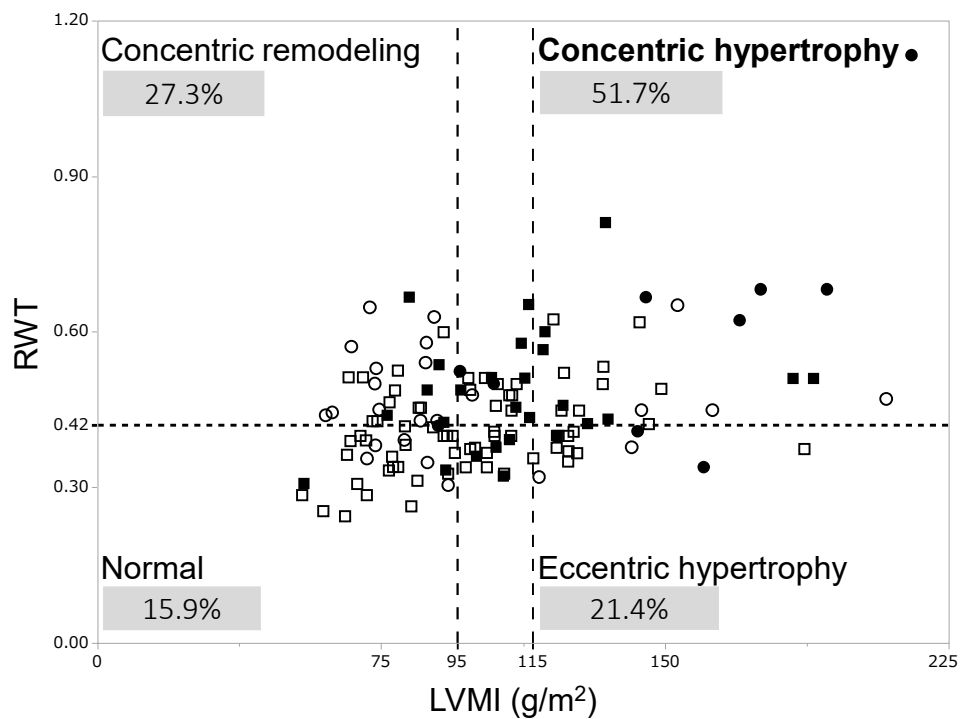

Supplement: Supplementary file 7 — Additional file 7 Fig. S1 Distributions of left ventricular mass index (LVMI) and relative wall thickness (RWT) in 131 patients are shown with information about mortality (A) and MACE incidence (B) in each category of LV geometry. Males and females are indicated as squares and circles, respectively. Patients with death (A) and MACE (B) are indicated by closed shapes. Mortality (A) and MACE incidence (B) in each category are shown. [file 12882_2021_2321_MOESM7_ESM.pdf]
